# 1918 Influenza Pandemic and Highly Conserved Viruses with Two Receptor-Binding Variants

**DOI:** 10.3201/eid0910.020789

**Published:** 2003-10

**Authors:** Ann H. Reid, Thomas A. Janczewski, Raina M. Lourens, Alex J. Elliot, Rod S. Daniels, Colin L. Berry, John S. Oxford, Jeffery K. Taubenberger

**Affiliations:** * Armed Forces Institute of Pathology, Rockville, Maryland, USA; † National Institute for Medical Research, London, United Kingdom; ‡ Queen Mary’s School of Medicine and Dentistry, London, United Kingdom; § Retroscreen Virology, Ltd., London, United Kingdom

## Abstract

The “Spanish influenza pandemic swept the globe in the autumn and winter of 1918–19, and resulted in the deaths of approximately 40 million people. Clinically, epidemiologically, and pathologically, the disease was remarkably uniform, which suggests that similar viruses were causing disease around the world. To assess the homogeneity of the 1918 pandemic influenza virus, partial hemagglutinin gene sequences have been determined for five cases, including two newly identified samples from London, United Kingdom. The strains show 98.9% to 99.8% nucleotide sequence identity. One of the few differences between the strains maps to the receptor-binding site of hemagglutinin, suggesting that two receptor-binding configurations were co-circulating during the pandemic. The results suggest that in the early stages of an influenza A pandemic, mutations that occur during replication do not become fixed so that a uniform “consensus” strain circulates for some time.

The 1918–19 influenza pandemic began, in some parts of the world, with mild outbreaks in the spring of 1918. In the fall of that year, a lethal wave swept the globe. Outbreaks occurred in early September in North America, Europe, and Africa and spread rapidly, so that the disease had peaked and declined worldwide by the end of December (*1*–*4*). Many areas had an additional wave of the disease in the early months of 1919. In most communities, the fall wave of the pandemic lasted approximately l month, with 25% to 30% of the population experiencing symptomatic disease. Clinically, epidemiologically, and pathologically, the disease was remarkably uniform, suggesting that similar viruses were causing disease worldwide (*5*). To assess the homogeneity of the 1918 pandemic influenza virus, partial hemagglutinin (HA) gene sequences were determined for strains from five cases, including two newly identified samples from London, United Kingdom. The strains show 98.9% to 99.8% nucleotide sequence identity. One of the few differences between the strains maps to the receptor-binding site of HA, which suggests that two receptor-binding configurations were co-circulating during the pandemic.

Influenza A virus is capable of rapid genetic change in mammals (*6*–*8*). Its polymerase complex lacks proofreading capability, such that one in five virus particles produced is likely to contain a change at one of its approximately 13,500 nt (*9*). If such a change provides the virus with a competitive advantage, that strain quickly replaces its predecessor. In humans, the need to escape preexisting immunity exerts positive selection pressure on changes in amino acids comprising the antigenic sites of the surface glycoproteins, HA and neuraminidase (NA) (*6*,*10*). The process of progressive change in the antigenic properties of the virus is called antigenic drift and results in the emergence of an antigenically distinct variant strain every 2–3 years. Between drift epidemics, the influenza virus appears to be antigenically uniform (*11*), but the degree of genetic uniformity has not been studied extensively.

In pandemic influenza, one or both of the virus’s surface proteins are replaced with proteins to which the human population has no preexisting immunity (*6*,*12*). The virus then spreads explosively, producing symptomatic infection in up to one third of most populations. During the rapid initial spread of a pandemic strain, little antigenic pressure on the virus exists. One might expect the genetic structure under these circumstances to be relatively constant. However, the degree of genetic identity among viral isolates during a pandemic is not known. Very few full-length HA sequences of viruses from the peaks of the 1957 and 1968 pandemics are available, and all of these viruses had been grown at least once in eggs before sequencing—a process that can select for an unpredictable number of sequence changes (*13*,*14*). Therefore, this study represents an initial attempt to measure the degree of genetic homogeneity of a pandemic virus. Since the sequences have been obtained directly from clinical material, they contain no sequence changes attributable to culture.

## Materials and Methods

### Patients and Samples

The genetic sequences encoding the HA1 domains of three 1918 influenza strains have been determined (*15*,*16*). Two of the strains came from U.S. soldiers who died on September 26, 1918: one in Camp Upton, New York, and one in Fort Jackson, South Carolina. The third came from an Inuit woman who died in mid-November 1918 in a remote village on the Seward Peninsula of Alaska.

To obtain further samples for analysis, we examined autopsy material of 14 patients who died in the fall and winter of 1918 to 1919. The material consisted of formalin-fixed, paraffin-embedded tissues, stained slides, and clinical records from the files of the Morbid Anatomy Department of the Royal London Hospital. The cases were preselected by histologic criteria for further analysis, and samples were taken from patients who died from acute influenza after clinical courses of <1 week (*16**–**18*). Of these 14 lung samples, 4 were positive for influenza RNA on subsequent molecular genetic analysis, but only 2 had sufficient material for HA1 sequencing. The first patient was a 50-year-old woman admitted to the hospital on November 12, 1918, with influenza and pneumonia. She died on November 13. The postmortem diagnosis was bronchopneumonia. The second patient was a 25-year-old man admitted to the hospital on February 13, 1919. He died on February 15 of influenza. The postmortem diagnosis was lobar pneumonia with toxemia.

## Methods

Sample preparation, reverse transcription, polymerase chain reaction (PCR), and sequencing were performed as described previously (*15*). (Primers used are available upon request.) PCR was performed from at least two separate reverse transcription reactions, and products from at least two PCR reactions were sequenced in each case to ensure accuracy and exclude amplification artifacts. Sequences used to evaluate the complexity of pandemic and epidemic influenza strains were obtained from the Influenza Sequence Database (available from: URL: http://www.flu.lanl.gov/).

## Results

The 563–bp fragments sequenced for this study, encoding the antigenic and receptor-binding sites of the HA1 domain (*19*–*21*), represent the most variable portion of the influenza genome (Figure). The London cases were designated A/London/1/1918 (H1N1) and A/London/1/1919 (H1N1). These two sequences, when compared to the three previously sequenced North American strains (*15*), differ from each other by 1 nt to 3 nt, showing a sequence identity of 98.9% to 99.8%.

**Figure:**
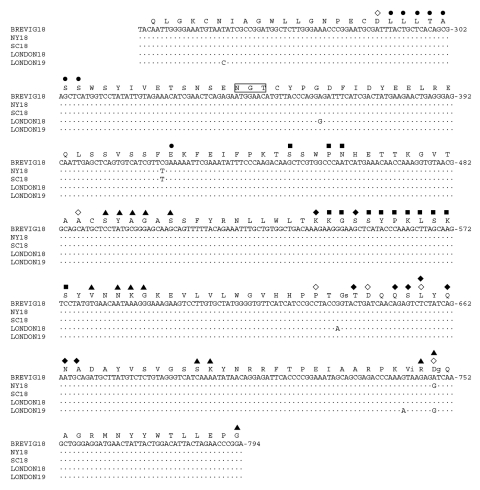
**Partial HA1 domain cDNA sequences from five 1918–19 cases.** A 563-bp fragment encoding antigenic (*19*,*20*) and receptor-binding (*21*) sites of the HA1 domain is shown, with the sequences aligned to A/Brevig Mission/1/1918 (BREVIG18) (*15*). Dots represent sequence identity as compared to BREVIG18. The numbering of the nucleotide sequence is aligned to A/PR/8/1934 (GenBank accession no. NC_002017) and refers to the sequence of the gene in the sense (mRNA) orientation. The partial HA1 translation product for BREVIG18 is shown above its cDNA sequence. Amino acid numbering is aligned to the H3 HA1 domain (*15*). Boxed amino acids indicate potential glycosylation sites as predicted by the sequence (*15*). Residues that have been shown experimentally to affect receptor-binding specificity in H1 HAs, D77, A138, P186, D190, L194, and D225 (*21*–*23*) are indicated by a ◇ symbol above these six residues. Residues defining four antigenic sites are indicated: C_b_ (●), S_a_ (■), S_b_ (◆), and C_a_ (▲) (*19*,*20*). Residues that have been mapped to both receptor-binding and antigenic sites (positions 194 and 225) are marked with two symbols. When a nucleotide change as compared to BREVIG18 results in a changed amino acid, the resultant amino acid is shown in lower case to the right of the BREVIG18 residue. Strain abbreviations and GenBank accession numbers: A/Brevig Mission/1/1918 (BREVIG18, # AF116575), A/South Carolina/1/1918 (SC18, # AF117241), A/New York/1/1918 (NY18, # AF116576), A/London/1/1918 (LONDON18, # AY184805), and A/London/1/1919 (LONDON19, # AY184806).

A/London/1/1918 shows 2 nt differences, compared to A/Brevig Mission/1/1918, one of which would change the amino acid at codon 188 from G to S (amino acid numbering is aligned to the H3 influenza HA). This residue is near several of the residues that have been shown experimentally to affect receptor-binding specificity of H1 HAs (*21*–*23*) and next to one of the mapped Sb antigenic site residues (*19*,*20*). A/London/1/1919 shows 3 nt differences from A/Brevig Mission/1/1918, 2 of which are nonsynonymous, resulting in changes of V223I and D225G. The V223I change is near Ca antigenic site residues, and the D225G change is at a residue that functions both in receptor-binding and as a Ca antigenic site residue. Amino acid 225 also varies among North American strains; A/New York/1/1918, like A/London/1/1919, has a glycine at position 225, as do most avian influenza strains. A/South Carolina/1/1918 and A/Brevig Mission/1/1918, like A/London/1/1918 and most subsequent human H1 strains, have aspartic acid at this position (Figure) (*15*). The relative genetic homogeneity of the 1918–19 isolates encouraged us to analyze sequences from the 1957 and 1968 pandemics.

GenBank contains complete HA1 domain–encoding sequences for eight 1957 H2N2 strains. As noted in previous studies of receptor-binding specificity (*22*,*24*), the 1957 strains have undergone varying passage histories, but all have been passed at least once. Three of the strains have been sequenced more than once and differ by as many as 8 nt within the same strain. Between sequences, the number of nucleotide differences ranges from only 1 nt difference between A/Chile/6/1957 and A/Davis/1/1957 to 12 differences between one of the A/Japan/305/1957 sequences and one of the A/Singapore/1/1957 sequences. Overall, the sequences show 98.9% to 99.9% identity at the nucleotide level, and 98.5% to 100% identity at the amino acid level.

More limited sequence data are available for the 1968 H3N2 pandemic strains. The complete HA1 domain sequence is available for only three strains, two of which have been sequenced twice each. The two A/NT/60/68/29C sequences differ by 4 nt. The most divergent sequences differ by 24 nt (A/NT/60/68/29C vs. A/Hong Kong/1/68), thus showing 97.6% to 100% identity between sequences at the nucleotide level, and 96.0% to 100% identity at the amino acid level.

Studies from epidemic years have yielded similar results. A 2001 study (*25*) examined variation in the HA gene of human H3N2 viruses in Spain from 1996 to 2000. During this time, strains antigenically similar to A/Wuhan/359/1995 were replaced by strains similar to A/Sydney/5/1997 and then by strains similar to A/Panama/2007/1999. Within the groups of viruses belonging to each antigenic group, sequence variation was minimal. For example, among the viruses that reacted antigenically with Sydney, but not Panama and Wuhan, 2–10 nt differences occurred over the 591 nt sequenced (98.3% to 99.7% identity).

An unpublished study provides sequences of the HA1 domain of the H3-subtype HA of 16 A/Sydney/05/1997-like (H3N2) influenza virus isolates circulating in Canada during the 1997/98 influenza epidemic season (GenBank no. AF087700–AF087702, AF087707, AF087708, AF096306–AF096316) (*26*). Two of the isolates had identical sequences, while the others varied by 1 nt to 14 nt over 981 nt (98.6% to 100% identity).

## Discussion

The three North American 1918 influenza strains sequenced previously were isolated from patients separated by nearly 2 months in time and almost 4,000 miles in distance (*27*). Two nucleotide differences were found among these three strains, one of which resulted in an amino acid substitution in the receptor-binding site (*15*). All three cases likely derived from the initial introduction of the fall wave into the United States, believed to have occurred in Boston in early September 1918. The virus then spread rapidly from Camp Devens, Massachusetts, the first U.S. army base to experience the epidemic, which then reached army bases throughout the eastern United States within 2 weeks (*2*). Influenza probably reached Brevig Mission, Alaska, via Seattle, Washington. The pandemic reached Camp Lewis, Washington, in mid-September, following the arrival of a troop ship from Philadelphia, Pennsylvania (*1*,*2*), and spread to Seattle by late September. After careful screening to exclude sick passengers, a ship left Seattle for Nome, Alaska, in mid-October, but days after its arrival local residents began falling ill (*1*). An account of the pandemic as it occurred in Brevig Mission reports that visitors from Nome brought the disease to the village in November (*28*). This chain of events suggests that the Alaskan outbreak was not the result of a separate introduction of the 1918 influenza from Asia to the West Coast of the United States.

The spring wave of the 1918 epidemic was widespread in France and Spain during April and May but did not reach England until June. The fall wave also arrived somewhat later in England than in continental Europe and the United States; peak mortality in London occurred during the first 2 weeks of November (*2*). A second peak occurred in the third week of February 1919. One strain from each of these peaks was sequenced for this study.

Our results show that strains separated by over 7,500 miles (Brevig Mission, Alaska, to London, United Kingdom) and several months (September 26, 1918, to February 15, 1919) share a sequence identity of 99%. This level of genetic homogeneity is slightly higher than that seen for the available 1957 and 1968 pandemic strains, but the 1957 and 1968 strains were not sequenced directly from clinical material. Sequences from different passages of the same strain were sometimes as different from each other as they were from other strains (*29*), suggesting that sequence heterogeneity observed was the result of culture adaptation, making it impossible to determine how homogeneous the pandemic viruses actually were. Even so, the 1957 and 1968 pandemic strains show >97% identity between strains. Similar levels of genetic homogeneity were seen in strains from case-patients isolated from a drift epidemic in 1997. Thus, influenza viruses circulating during a single outbreak, whether epidemic or pandemic, show levels of sequence identity consistent with the uniformity of the 1918 cases.

Despite the uniformity of the 1918 strains, one of the variable sites is an amino acid known to be important in receptor binding (*21*). At a subset of amino acids critical for receptor binding, avian strains differ from swine H1s at only one amino acid, E190D (*15*). At these amino acids, two of the cases (A/New York/1/1918 and A/London/1/1919) are identical to that of A/sw/Iowa/1976/31 (a classical swine strain). The other 1918 cases have an additional change from the avian consensus at amino acid 225. Since swine viruses with the same receptor site as A/sw/Iowa/1976/31 bind both SAα2,3Gal and SAα2,6Gal (*14*), A/New York/1/1918 and A/London/1/1919 probably also had the capacity to bind both receptors. Because two of five 1918–19 analyzed fall wave strains from case-patients have the swine-like receptor-binding pattern, the E190D change alone is apparently sufficient to allow viral replication in the human respiratory tract. However, the existence of three strains with the additional G225D change shows that both receptor-binding variants were co-circulating throughout the pandemic. The current evidence does not suggest progression from one receptor-binding pattern to the other during the pandemic, since the two variants are present, on both continents, both early and late in the pandemic. Since residue 225 has also been identified as part of the Ca antigenic site (*19*), the co-circulating strains possibly differed in antigenic reactivity as well as receptor-binding characteristics.

This study is the first to examine the genetic homogeneity of a pandemic influenza virus directly from clinical material. The results suggest that in the early stages of a pandemic, mutations that occur during replication do not become fixed so that a uniform consensus strain circulates for some time. Studies of influenza strains circulating after 1919 should provide insight into how pandemic viruses evolve after the initial waves through immunologically vulnerable populations. In terms of pandemic planning, our results indicate that a specific antiviral drug or vaccine would have a uniform effect during the important and often lethal first wave of a pandemic (*30*,*31*).
